# High prevalence of small *Babesia* species in canines of Kerala, South India

**DOI:** 10.14202/vetworld.2017.1319-1323

**Published:** 2017-11-09

**Authors:** Kollannur Jose Jain, Bindu Lakshmanan, Karunakaran Syamala, Jose E Praveena, Thazhathuveetil Aravindakshan

**Affiliations:** 1 School of Applied Animal Production and Biotechnology, College of Veterinary and Animal Sciences, Kerala Veterinary and Animal Sciences University, Mannuthy, Thrissur - 680 651, Kerala, India; 2 Department of Veterinary Parasitology, College of Veterinary and Animal Sciences, Kerala Veterinary and Animal Sciences University, Mannuthy, Thrissur - 680 651, Kerala, India

**Keywords:** 18S rRNA gene, *Babesia gibsoni*, dogs, hematology, phylogeny, polymerase chain reaction

## Abstract

**Aim:**

Canine babesiosis is an important vector-borne hemoparasitic disease caused by *Babesia canis vogeli* and *Babesia gibsoni*, in India. The communication places on record the salient findings of the study directed to detect and characterize the pathogenic *B. gibsoni* isolates of Kerala state.

**Materials and Methods::**

A total of 150 dogs were examined for the presence of hemoparasites by light microscopy as well as by PCR targeting the 18S rRNA gene of *B. gibsoni*. Hematological parameters were also analysed. Phylogenetic tree was constructed based on Tamura kei model adopting ML method.

**Results::**

A sensitive and specific polymerase chain reaction assay was developed with newly designed primer pair BAGI-F/BAGI-R for the amplification of 488 bp fragment of 18S rRNA gene of *B. gibsoni*. Out of the 150 dogs examined, molecular evidence of *B. gibsoni* was recorded in 47.3% animals, while light microscopy detected the infection in 26.67% cases. The phylogenetic analyses revealed that *B. gibsoni*, Kerala, isolate was closest and occurred together with Bareilly isolate. Anemia and thrombocytopenia were the significant hematological alterations in chronic *B. gibsoni* infection.

**Conclusion::**

A high prevalence of natural infection of *B. gibsoni* was detected among the study population. The affected animals showed anaemia and thrombocytopenia. Phylogenetic analysis of this pathogenic isolate from south India revealed the closest similarity with Bareilly isolates.

## Introduction

Babesiosis is one among the most prevalent tick-borne parasitic diseases of dogs in south India, caused by either *Babesia gibsoni*, the small piroplasm or *Babesia canis*, the large piroplasm. Canine babesiosis is exhibited in a wide range of presentations from subclinical disease to serious illness characterized by fever, pallor, anemia, jaundice, splenomegaly, weakness and collapse associated with intra- and extra-vascular hemolysis. The large piroplasm is classified into three different phylogenetic groups referred to as subspecies *Babesia canis, Babesia canis vogeli*, and *Babesia canis rossi*, of which *B. canis vogeli* is reported from India. The small *Babesia* piroplasm in dogs was reported for the first time in India in 1910 [1]. Since then, there exist sporadic reports on microscopic detection of *B. gibsoni* in India [2-8]. Although *B. gibsoni* is mainly transmitted by *Haemaphysalis longicornis* and *Rhipicephalus sanguineus* ticks [9], the former was reported to be the putative vector in India [10].

Morphologically, *B. gibsoni* organisms are small (1-2.5 µm) occurring as ovoid or annular forms within the erythrocytes, but three distinct isolates of these piroplasms were reported based on analysis of 18S rRNA gene of the isolates from Asia, United Sates, Africa, and Spain [11]. Nevertheless, molecular detection of the organism has been attempted by very few workers in the country [12-15]. Apart from *B. gibsoni*, small intraerythrocytic piroplasms such as *Theileria equi*, *Theileria annulata*, and an un named *Theileria* spp. were also reported from dogs in Spain and South Africa [16,17]. Genetic characterization of the small *Babesia* spp. isolated from four north and north eastern states of India, established ITS-1 and 18S rRNA genes to be useful genetic markers to study the evolutionary relationship of *B. gibsoni* isolates and reported the existence of atypical *Babesia* spp. in dogs in the region [13].

The present investigation is directed toward identifying the prevalence of *B. gibsoni* infection in Kerala, South India using a single step polymerase chain reaction (PCR) protocol and analyzing the phylogeny of the South Indian isolates based on 18S rRNA gene sequence. The hematological parameters of PCR positive animals are also evaluated in this study to identify the health status of dogs during subclinical infection.

## Materials and Methods

### Ethical approval

No ethical approval was required as the samples were collected from animals presented to the different hospitals for treatment/regular checkup.

### Sample collection

A total number of 150 client-owned dogs were included in the study. Peripheral blood smears were prepared from the ear tips and stained with Giemsa to identify the presence of hemoparasites by light microscopy. Whole blood samples were collected from saphenous vein in 6 ml K2 EDTA coated vacutainer under aseptic conditions for DNA extraction and estimation of hematological parameters.

### Hematological parameters

The hematological parameters of dogs were analyzed using autoanalyzer (Master T, Hospitex international, Italy) and compared with the normal range. Parameters such as red blood cell (RBC) count, white blood cell count, hemoglobin (Hb), hematocrit, (HCT), mean corpuscular volume (MCV), red cell distribution width (RDW), and platelet (PLT) count were estimated.

### Isolation of genomic DNA

Genomic DNA extraction was performed by phenol–chloroform method [18] with modifications [19]. The quality of DNA in the final elutes were estimated using nano spectrophotometer (Nanodrop 200 C, Thermo Scientific, USA). Samples which yielded a ratio between 1.7 and 1.9 at 260:280 nm were selected for analysis.

### PCR

The PCR was initially standardized by gradient thermal protocol using known positive blood DNA sample as revealed by heavy parasitemia during microscopic examination of stained blood smears. The primers targeting a 488 bp region of 18S rRNA gene of *B. gibsoni* were designed using Primer 3 software (www.bioinfo.ut.ee>primer3-0.4.0) based on the corresponding gene sequences of *B. gibsoni* isolate available in the GenBank. The suitability was checked with sequence manipulation suite software (www.bioinformatics.org>sms2) and specificity confirmed by blast analysis (BLASTntool: www.ncbi.nlm.nih.gov).

Gradient PCR was performed in a 25 µL reaction volume containing 2.5 µL of buffer (10×) without MgCl_2_, 200 µM each of dNTP, 25 pmol each of forward (BAGI-F 5’- TTG GCG GCG TTT ATT AGT TC-3’) and reverse primers (BAGI-R -5’ AAA GGG GAA AAC CCC AAA AG-3’), 2.0 mM of MgCl_2_, 1.5U of *Taq* DNA polymerase, and 5.0 µL of template DNA. All the reagents were procured from Sigma-Aldrich (USA). A no template control was included in each run. A gradient thermal cycling program (Bio-Rad T100, USA) with initial denaturation at 94°C for 5 min followed by 35 cycles of denaturation (94°C, 30 s), annealing (54°C to 61°C, 30 s) and extension (72°C, 1 min), and a final extension at 72°C for 5 min was adopted. The amplified PCR products were subjected to electrophoresis in 1.5% agarose gel (Hoefer, USA) and visualized in Gel-documentation system (Bio-Rad Laboratories, USA).

### Sequencing and phylogenetic analysis

The amplicons were purified using silica gel purification columns (GeneJET, Thermo scientific), sequenced using Sanger’s dideoxy chain termination method and the sequences aligned using Sequencher Version 5.0 (SciGenom Labs Pvt Ltd, Cochin). Bidirectional sequencing was done with both forward and reverse primers. The sequences were aligned using EMBOSS (www.bioinformatics.nl>cgi-bin>merger) and blasted using NCBI BLAST tool (www.blast.ncbi.nlm.nih.gov>blast) to analyze their similarity with other published sequences available in online databases. Sequences corresponding to partial 18S rRNA gene of *B. gibsoni* were downloaded from online databases and aligned with the corresponding partial sequence of *B. gibsoni* obtained in this study and were further utilized for the construction of phylogenetic tree using maximum likelihood method. The bootstrap consensus tree was inferred from 1000 replicates, in Dambe 5.5.24.

## Results

**Figure-1:**
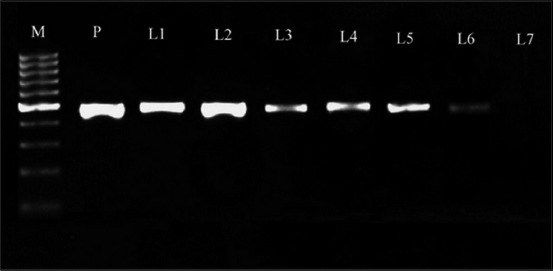
Amplicons of 18S rRNA gene of *Babesia gibsoni*. Lanes M: 100 bp DNA ladder, P: Positive control with 488 bp amplicons, 1-7: serially diluted templates (10^−1^ to 10^−7^)

PCR was standardized for amplifying partial region of 18S rRNA gene of *B. gibsoni* by gradient protocol using newly designed primers (BAGI-F and BAGI-R). A bright band of size between 400 and 500 bp was observed in all annealing temperatures between 54°C and 61°C using known positive control DNA. Alignment of the bidirectional nucleotide sequences of the product yielded a 488 bp sequence which revealed 99% similarity with corresponding published sequences of *B. gibsoni*. There was no significant similarity with any other species of *Babesia*, thus confirming the identity of the small intraerythrocytic piroplasms of this genus among dogs in Kerala as *B. gibsoni*. The sequence was submitted to GenBank and assigned with accession number KX766396. The specificity of the designed primers was also well ascertained by the absence of cross-amplification with DNA of common hemoparasites in south India, namely, *B. canis*, *Ehrlichia canis, Trypanosoma evansi*, or *Dirofilaria* spp. available in the Department of Veterinary Parasitology of this institution. Sensitivity of the primers was determined based on the amplification of serially diluted DNA of known positive controls amplified at an annealing temperature of 59°C. Distinct amplicons were observed up to 10^−6^ dilution of template DNA (2.935 pg DNA) (Figure-1).

Out of the 150 dogs, 71 (47.33%) were found to be PCR positive for *B. gibsoni*, while only 40 were blood smear positive. The higher prevalence detected by PCR indicated the presence of subclinical/chronic status of infection among a significant proportion of study population.

**Figure-2:**
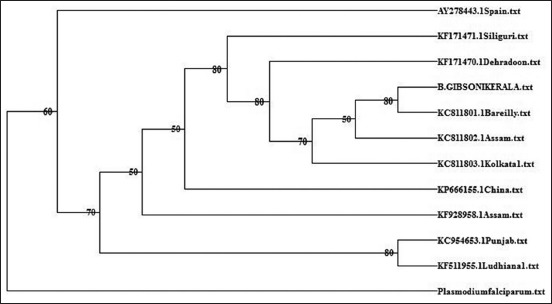
Phylogenetic analysis of 18S rRNA gene of *Babesia gibsoni*.

The phylogenetic tree based on maximum likelihood method with Tamura-Nei model using 18S rRNA gene sequences with *Plasmodium falciparum* as outgroup species is presented in Figure-2. Phylogenetic analyses revealed that Kerala isolate of *B. gibsoni* was closest with Bareilly isolate (KC811801) and occurred as a single clade. This clade was closest and could be observed as a sister clade of other Indian isolates, i.e., Assam isolate (KC 811802) and Kolkata isolate (KC 811803). However, the Kerala isolate was farthest from the Punjab (KC 954653) to Ludhiana isolate (KF 511955).

The hematological parameters of dogs that were PCR positive for *B. gibsoni* without any evidence of parasitemia in stained peripheral blood smear were analyzed (Table-1). The results suggested that the PCR positive animals exhibited a near normal leukogram while the erythrogram values were below the normal range. The hematological alterations of decreased RBC, Hb, and HCT suggested that all animals were anemic. Anisocytosis was suggested by the high values of RDW. Severe thrombocytopenia was also observed in *B. gibsoni* positive animals.

**Table-1:** Hematological parameters.

Parameters	Minimum	Maximum	Mean±SE	Normal
WBC (10^3^/µl)	5.2	38.4	16.26±4.00	6-17
LYM%	6.3	62.8	22.95±6.401	12-30
MON%	1.3	12.5	4.95±1.259	3-10
GRA%	34.6	92.4	72.1±6.711	60-74
RBC (10^6^/µl)	0.46	4.04	2.84±0.497	5.5-8.5
Hb (g/dl)	1.3	11.9	7.57±1.45	12-18
HCT (%)	3.5	28.8	18.625±3.249	37-55
MCV (µm^3^)	55	76.1	66.97±2.633	60-77
MCH (pg)	15.8	32.1	26.8±1.9361	19-25
MCHC (g/dl)	26.8	51.5	40±2.64	30-36
RDW (%)	11.1	19.2	15.37±1.123	11-14
PLT (10^3^/µl)	19	89	50.62±8.333	160-525
MPV (µm^3^)	5	8.1	6.37±0.3282	7-13
PCT (%)	0.011	0.058	0.033±0.0060	0.150-0.390

WBC=White blood cell count, RBC=Red blood cell, Hb=Hemoglobin, HCT=Hematocrit, MCV=Mean corpuscular volume, RDW=Red cell distribution width, PLT=Platelet, MCH=Mean corpuscular hemoglobin, MCHC=Mean corpuscular hemoglobin concentration, MPV=Mean platelet volume, PCT=Plateletcrit

## Discussion

A sensitive and specific PCR protocol for detection of *B. gibsoni* was standardized using novel primers. The primers could detect infection with low as 2.935 pg template DNA in ethidium bromide stained gels without any nonspecific products. This also suggests the suitability of the protocol for assessing the clearance after chemotherapy, which is especially important with *B. gibsoni*, as combinations therapies to eliminate this infection were mostly encountered with either remission or low recovery rates [20,21]. The absence of amplification with the DNA of the most common hemoparasites of dogs in Kerala established the specificity of the primer and the protocol. A significant proportion of dogs (47.33%) were found infected with *B. gibsoni* piroplasm using PCR when compared to conventional microscopy. This clearly demonstrated that PCR is more sensitive than light microscopic examination of stained blood smear [22]. The higher prevalence detected by PCR indicated the presence of subclinical/chronic status of infection among a significant proportion of study population. Jefferies *et al*. [23] had also observed that chronic phase of *B. gibsoni* infection fail to reveal parasitemia in blood smear and could be confirmed by PCR. Subsequent to the first report on natural infections of *B. gibsoni* in dogs in Kerala [5], the decade has witnessed a sharp rise in the prevalence of the organism in the state. The reports of the incidence of *B*. *gibsoni* among dogs in the state were based on the results of conventional microscopy until [24] provided molecular evidence of *B. gibsoni* in dogs in Thrissur following the protocol of Birkenheuer *et al*. [25] utilizing semi-nested PCR targeting the 18S rRNA gene. Reports suggest that *B. gibsoni* infection was predominant in dogs in North India [26] and in Chennai [27] while the prevalence of the organism was lower than *B. canis* in Pakistan [28]. The increased prevalence in the recent years in Kerala could be the result of complex interaction of factors such as climate, habitat management change, increased tick abundance, and distribution. This has necessitated molecular characterization of the small piroplasms of the state.

Molecular characterization of *B. gibsoni* based on 18S rRNA gene sequence was effective in differentiating the various genotypes of the organism [13,29]. Apart from employing small subunit ribosomal RNA gene sequence, heat shock protein [30] and *B. gibsoni* thrombospondin-related adhesive protein [31] were also reported to be useful for phylogenetic analysis of *B. gibsoni*. Sequencing of the PCR product obtained with newly designed primer pairs (BAGIF and BAGIR) yielded a 488 bp sequence of 18S r RNA gene which revealed 99% similarity with several corresponding sequences of *B. gibsoni* available in online databases. The phylogenetic tree based on partial 18S rRNA gene revealed that Kerala isolate was closest to Bareilly isolate (*B. gibsoni* Asian genotype) and farthest from the two isolates from North Indian states, namely, Punjab and Ludhiana. Singh *et al*. [31] had observed that *B. gibsoni* endemic in dogs in India and Bangladesh formed a distinct genetic clade.

Animals that were PCR positive without any intraerythrocytic organisms being detectable during blood smear examination were targeted for the analysis of hematological parameters, to provide information on the subclinical/chronic status of *B. gibsoni* among naturally infected dogs. The comparison of mean values with the normal range reported by Meinkoth *et al*. [32], revealed that anemia and thrombocytopenia were evident in chronic infections of *B. gibsoni*. Hemolysis of infected erythrocytes due to direct parasite-induced damage has been a well-documented finding of canine babesiosis [33]. The unaltered MCV demonstrated a normocytic anemia. Increased RDW indicated anisocytosis and was considered more sensitive than MCV in detecting early changes in theses cell populations [34]. Anemic changes despite detectable parasitemia correlated with the observations of Brown *et al*. [35], who suggested that severity of anemia was seldom proportional to magnitude of the peripheral parasitemia and oxidative and immune-mediated injury also contributed to the erythrocyte loss. Thrombocytopenia in *B. gibsoni* infection was associated with an immune-mediated phenomenon involving the binding of IgG to the PLT surface followed by removal of PLTs [20,36] or due to the release of inflammatory mediators during RBC lysis [37]. In this context, significant hematological alterations in the absence of detectable parasites in conventional microscopy throws light on the pathogenicity of chronic infections of small *Babesia* species which demands the use of sensitive PCR protocol for quick detection of the infection to select timely treatment options. Future investigations need be directed toward exploring the possibility of these hematologic alterations to be used as phenotypic markers of *B. gibsoni* infection which could be adopted infield practice. In the light of increasing prevalence of this species in this state, there is an urgent need to evaluate the genotypes of this isolates to further explore the molecular epidemiology of this infection as well as to assess the novel therapeutic protocols for complete clearance of parasite in the hosts.

## Conclusion

The increasing prevalence of small *Babesia* piroplasm in Kerala, South India, was documented with the aid of a sensitive and specific PCR protocol amplifying a 488 bp sequence of 18S rRNA gene of *B. gibsoni*. The Kerala isolate shared closest evolutionary relationship with a North Indian (Bareilly) isolate which was reported as Asian genotype. The protocol could effectively amplify the specific gene fragment during chronic/subclinical phase of infection. This phase of infection was characterized by severe anemia, anisocytosis, thrombocytopenia, and a near normal leukogram even without detectable parasitemia in peripheral blood smears suggesting the high pathogenicity of the parasite. The alarmingly high prevalence of pathogenic small *Babesia* among canines of the state necessitates further trials to validate novel treatment modules and genotype variations.

## Authors’ Contributions

KJJ and JEP have performed the molecular part of the work, BL has designed the study and drafted the manuscript, KS has performed hematological analysis and TA has critically reviewed the manuscript. All authors have read and approved the final manuscript.
